# Genotypic and Phenotypic Characterization of *Cronobacter* spp. Strains Isolated from Powdered Milk Formulas and Dairy Production Environments

**DOI:** 10.3390/microorganisms14030593

**Published:** 2026-03-06

**Authors:** Julio Parra-Flores, Beatriz Daza-Prieto, Miriam Troncoso, Guillermo Figueroa, Maria I. Reyes-Fuentes, Ondrej Holy, Ariadnna Cruz-Córdova, Werner Ruppitsch, Stephen Forsythe

**Affiliations:** 1 Department of Nutrition and Public Health, Facultad de Ciencias de la Salud y de los Alimentos, Universidad del Bío-Bío, Chillán 3800708, Chile; 2 Laboratorio de Investigación Celular y Molecular en Ciencias de la Salud, Facultad de Ciencias de la Salud y de los Alimentos, Universidad del Bío-Bío, Chillán 3800708, Chile; maria.reyes.f@ug.uchile.cl; 3 Institute of Medical Microbiology and Hygiene, Austrian Agency for Health and Food Safety, 1220 Vienna, Austria; beatriz.daza-prieto@ages.at; 4 Fundación Instituto Profesional Duoc UC, Santiago 8240000, Chile; mir.troncoso@profesor.duoc.cl; 5 Microbiology and Probiotics Laboratory, Institute of Nutrition and Food Technology, Universidad de Chile, Santiago 7830490, Chile; 6 Department of Health Care Sciences, Faculty of Humanities, Tomas Bata University in Zlín, 76001 Zlín, Czech Republic; holy@utb.cz; 7 Immunochemistry Research Laboratory, Hospital Infantil de México Federico Gómez, Mexico City 06720, Mexico; ariadnnacruz@yahoo.com.mx; 8 Institute of Hygiene and Medical Microbiology, Medical University Innsbruck, 6020 Innsbruck, Austria; werner.ruppitsch@i-med.ac.at; 9 FoodMicrobe.com Ltd., Keyworth, Nottinghamshire NG12 5GY, UK

**Keywords:** *Cronobacter sakazakii*, *Cronobacter malonaticus*, WGS, powdered milk, dairy production environment

## Abstract

*Cronobacter* spp. is a pathogenic genus comprising seven species, of which *C. sakazakii* is particularly notable for its association with neonatal outbreaks linked to powdered infant formula. The severity of infections is associated with virulence factors (VFs) and β-lactam antibiotic resistance genes (ARGs). Next-generation sequencing (NGS) has enabled precise strain typing through core genome multilocus sequence typing (cgMLST), enhancing discrimination and accuracy. This study aimed to use cgMLST (2831 genes) to genomically characterize 34 *Cronobacter* strains which had been isolated from powdered milk and production surfaces between 2011 and 2022. The identified strains included *C. sakazakii* ST1, ST4, ST13, ST31 and ST83, as well as *C. malonaticus* ST60. Overall, there were eight clusters of closely related strains. All strains exhibited resistance to cephalothin, 18 were resistant to ceftazidime and 11 to ampicillin. Various resistance genes (*bla_CSA_*, *bla_CMA_*, *fos*, *qacJ*, *marA*, *AcrAB-TolC*, and *mcr-9*.*1*) and virulence genes (*cpa*, *nanAKT*, *fic*, *relB*, *fliC*) were detected, with some genes being exclusive to *C. sakazakii*. All strains carried plasmids and mobile genetic elements. The multidrug resistance and presence of virulence genes in these isolates highlight the significant risk that *C. sakazakii*-contaminated powdered dairy products pose to public health, underscoring the need to adopt proper hygienic manufacturing practices and effectively implement HACCP in their production.

## 1. Introduction

*Cronobacter* is a genus of bacterial pathogens consisting of seven species: *C. sakazakii*, *C. malonaticus*, *C. universalis*, *C. turicensis*, *C. muytjensii*, *C. dublinensis*, and *C. condimenti* [1,2,3]. The species of highest clinical significance is *C. sakazakii*, which has been reported in cases and outbreaks associated with infants fed contaminated powdered infant formula, followed by *C. malonaticus* in adult infections [4,5,6].

The main clinical symptoms associated with *C. sakazakii* include meningitis, septicemia, or necrotizing enteritis in infants [7,8], while for *C. malonaticus*, symptoms are associated with gastroenteritis and urinary tract infections [9]. Documented mortality rates range from 15% to 80% for general infections and 15% to 25% for neonatal meningitis and septicemia, respectively [10].

The disease is associated with the consumption of rehydrated powdered formula, which acts as a source of the pathogen, as well as with utensils and equipment serving as reservoirs. *Cronobacter* spp. can be isolated from powdered infant formulas, rehydrated milk, cereals, foods, water, surfaces, homes, and hospitals [11,12]. Although the source of contamination is not always identified, it is suggested that manufacturing plants for powdered infant formula (PIF) are its natural habitat [4,13]. Controlling *Cronobacter* spp. in the early stages of PIF production is essential to reduce the incidence of pathogens, as some strains have been found up to two years after packaging [14].

Between 1958 and 2016, eight countries reported cases of *C. sakazakii*, raising growing concerns for public health [15]. In the United States, between 2002 and 2024, 23 cases of *C. sakazakii* in infants were identified and confirmed, of which 8 were invasive and 15 non-invasive. The invasive cases were associated with a younger infant population, with a median age of 18 days, compared to 98 days for the non-invasive cases. Open and closed powdered infant formulas (PIFs) were cultured from 10 investigations representing both invasive and non-invasive cases. The open PIF samples were analyzed, and 25% tested positive for *C. sakazakii* ST4, while one (11%) closed PIF sample also tested positive for *C. sakazakii* ST4 [16].

Molecular typing has established itself as an essential approach for understanding bacteria that colonize specific ecological niches. The implementation of the multilocus sequence typing (MLST) scheme developed by Baldwin et al. (2009) has enhanced the identification of *Cronobacter* spp., providing valuable information on the genetic prevalence of *C. sakazakii* associated with neonatal meningitis in infants [4,17]. With the broader use of whole genome sequencing (WGS), detailed gene-by-gene analysis, using MLST typing, especially core genome MLST (cgMLST), offers high discrimination and a more accurate view of the taxonomic differences among pathogenic strains. Additionally, WGS has facilitated the identification of pathogens and the detection of genes related to antibiotic resistance, virulence factors, and the presence of plasmids, enabling the establishment of more precise epidemiological relationships [18,19]. In this context, both resistance and virulence are crucial factors in the pathogenicity of *Cronobacter* spp., and methods such as MLST, cgMLST, and WGS are fundamental for the accurate characterization of these strains, contributing to a better understanding of their epidemiology and control strategies.

Given the risk to infant health, there is a need to generate more information concerning *C. sakazakii*’s presence in powdered milk formulas and the production environment. Therefore, the aim of this study is to conduct the genotypic and phenotypic characterization of *Cronobacter* spp. strains isolated from powdered milk formulas and dairy production environments.

## 2. Materials and Methods

### 2.1. Isolation and Identification

This study utilized a total of 34 strains that were exclusively isolated from powdered milk, ingredients (milk powdered ingredients and milk serum) and the manufacturing environments of a single dairy formula production company between 2011 and 2022 in Chile. *Cronobacter* spp. strains were isolated using the method outlined by Iversen et al. [20]. Samples from food and the environment were initially pre-enriched in buffered peptone water (BPW), followed by enrichment in Enterobacteriaceae broth (BD Difco, Sparks, MD, USA), and then plated on Brilliance CM 1035 chromogenic agar (Oxoid Thermo-Fisher, UK). They were subsequently purified on trypticase soy agar (BD Difco, Sparks, MD, USA). Before sequencing, strains were identified by matrix-assisted laser desorption ionization–time of flight mass spectrometry (MALDI-TOF MS) using the MBT Compass IVD 4.1.60 software (Bruker, Billerica, MA, USA) as described by Lepuschitz et al. [21]. Identification of *Cronobacter* spp. strains was further complemented with ribosomal multilocus sequence typing (rMLST) software available at https://pubmlst.org/species-id (accessed on 15 January 2026) [22].

### 2.2. Whole Genome Sequencing (WGS)

*Cronobacter* spp. isolates were cultivated on Columbia blood agar plates (bioMérieux, Marcy-l’Étoile, France) at 37 °C for 24 h. For whole genome sequencing (WGS), DNA was extracted using the MagAttract HMW DNA Kit (Qiagen, Hilden, Germany). DNA quantification was performed with a Qubit 2.0 fluorometer (Thermo Fisher Scientific, Waltham, MA, USA). Sequencing libraries were prepared using Nextera XT chemistry for paired-end sequencing (2 × 300 bp) on an Illumina MiSeq sequencer, targeting a minimum of 80-fold coverage. The resulting FASTQ files were quality trimmed and de novo assembled with SPAdes v. 3.11.1. Contigs underwent filtering for a minimum coverage of 5× and a length of 200 bp using MBioSEQ Ridom Typer 11.1 software (Ridom, Münster, Germany) [23]. Raw reads were controlled for quality with FastQC, and Trimmomatic was used to remove adapter sequences and low-quality reads.

### 2.3. Sequence Type (ST) and Core Genome Multilocus Sequence Typing (cgMLST) of Cronobacter *spp*.

A total of 2831 targets were used to establish the core genome multilocus sequence typing (cgMLST) scheme of *Cronobacter* spp. using strain ATCC BAA-894 as a reference using a target gene loci task template of the MBioSEQ Ridom Typer 11.1 software (Ridom, Münster, Germany) [21,23]. According to the determined cgMLST scheme, isolates were visualized with a minimum spanning tree (MST) to establish their genotypic relationships and defining as clusters those isolates with maximum differences of 10 alleles [21]. In addition, the sequences of the seven housekeeping genes of the conventional MLST for *Cronobacter sakazakii* (*atpD*, *fusA*, *glnS*, *gltB*, *gyrB*, *infB* and *ppsA*) were extracted and cross-checked against the *Cronobacter* MLST database https://pubmlst.org/organisms/cronobacter-spp (accessed on 15 January 2026) [17].

### 2.4. Determination of Serotypes

The *gnd* and *galF* genes profiles, specific to the serotype O region of *Cronobacter* spp., were analyzed through WGS using the BIGSdb tool provided in the PubMLST database https://pubmlst.org/organisms/cronobacter-spp (accessed on 15 January 2026).

### 2.5. Antibiotic Susceptibility

To assess antibiotic susceptibility, the disk diffusion method was employed following the guidelines set by the Clinical and Laboratory Standards Institute [24]. The commercial disks used included ampicillin (10 μg), amoxicillin–clavulanic acid (20/10 µg), ceftazidime (30 µg), ciprofloxacin (5 μg), chloramphenicol (30 μg), cefotaxime (30 μg), gentamicin (10 μg), cephalothin (30 μg), tetracycline (30 μg), and nalidixic acid (30 μg). The resistance and susceptibility profiles were characterized according to the manufacturer’s instructions. Strains of *Escherichia coli* ATCC 25922 and *Pseudomonas aeruginosa* ATCC 27853 served as internal controls.

### 2.6. Detection of Antibiotic Resistance and Virulence Genes

For the analysis of resistance genes, the Comprehensive Antibiotic Resistance Database (CARD) was utilized with the “perfect” and “strict” default settings, applying only high-quality and high-coverage sequences [25]. This was complemented by the Task Template AMRFinderPlus 4.0.15 available in the MBioSEQ Ridom Typer 11.1 software, using a method that ensures 100% identity and aligned overlap [26]. To identify genes and proteins associated with virulence, a manual search for virulence genes was conducted using the Gene Comparator tool available in PubMLST *Cronobacter*, applying a core threshold of 90% and a minimum identity of 70% https://pubmlst.org/bigsdb?db=pubmlst_cronobacter_isolates&page=query&genomes=1 (accessed on 16 January 2026).

### 2.7. Reconstruction of Plasmids, Chromosome, and Mobile Genetic Elements

The reconstruction of chromosomes, plasmids and mobile elements used the “Chromosome and Plasmids Overview” Task Template with the MOB-suite tool v3.1.8 [27]. For the identification of integrative mobile genetic elements (iMGEs) in WGS sequences, the “CGE MobileElementFinder” Task Template was employed along with the MobileElementFinder v1.1.2 tool with identify filter of 90% and aligned filter of 95% [28]. All Task Templates were executed in the MBioSEQ Ridom Typer 11.1 software [23].

## 3. Results

### 3.1. Isolation and Identification

Of the 34 strains, 5 (14.7%) had been isolated from powdered infant formula (PIF), 4 (11.8%) from powdered milk (PM), 9 (26.5%) from ingredients, and 16 (47.0%) from surfaces and environments. The year 2016 had the highest number of isolates, with 16 strains, followed by 2017 with 7 strains. Among the 34 strains analyzed, 31 were confirmed as *Cronobacter sakazakii* and 3 as *Cronobacter malonaticus* (Table 1).

### 3.2. Sequence Type (ST) and Core Genome Multilocus Sequence Typing (cgMLST) of Cronobacter *spp*.

The majority (91.2%, 31/34) of the isolates were confirmed as *C. sakazakii*. Of these, 8 isolates were genotyped as ST1 (CC1 and *CsakO1*), 1 as ST4 (CC4 and *CsakO4*), 2 as ST13 (CC13 and *CsakO2*), 8 strains as ST31 (CC31 and *CsakO2*), and 12 strains as ST83 (CC83 and *CsakO7*). The remaining 8.8% (3/34) were identified as *C. malonaticus* ST60 (CC60 and *CmalO1*) (Table 1).

**Figure 1:**
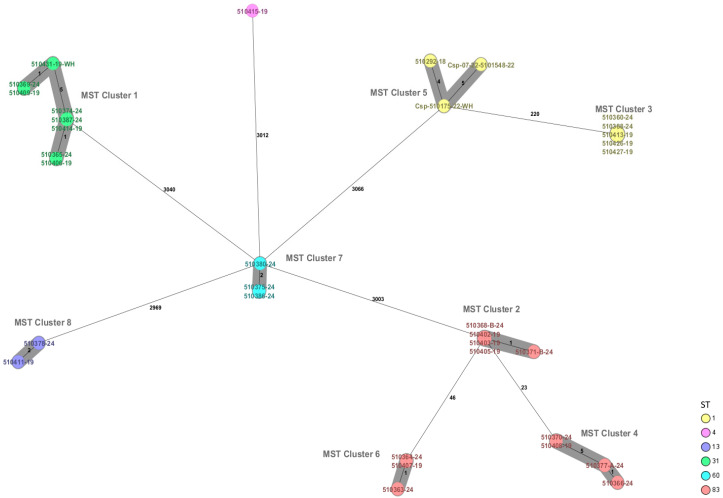
Minimum spanning tree (MST) of the thirty-one *Cronobacter sakazakii* and three *Cronobacter malonaticus* isolates in this study. Isolates are represented as colored circles according to their sequence type (ST) as defined using the 7-loci MLST scheme. Black numbers on the connection lines indicate the number of allelic differences between isolates from the cgMLST scheme comprising 2831 target genes for *Cronobacter sakazakii*/*C. malonaticus*. Isolates falling under the cluster threshold of 10 alleles are marked in grey as clusters.

Using the gene-by-gene cgMLST scheme, the isolates were grouped into eight closely related clusters. *C. sakazakii* ST1 was divided into two clusters: cluster number 3, which includes five isolates with no allele differences, and cluster number 5, which contains three isolates with a maximum of five allele differences. *C. sakazakii* ST13 was grouped in cluster number 8 with two strains and two allele differences, while ST31 was placed in cluster number 1 with eight isolates, showing between one and five allele differences. *C. sakazakii* ST83 was grouped into three clusters: cluster number 2 with five strains and one allele difference, in cluster number 4 with four strains and five allele differences, and in cluster number 6 with three strains and one allele difference. *C. malonaticus* was grouped in cluster number 7 with three strains and two allele differences (Figure 1).

**Figure 2:**
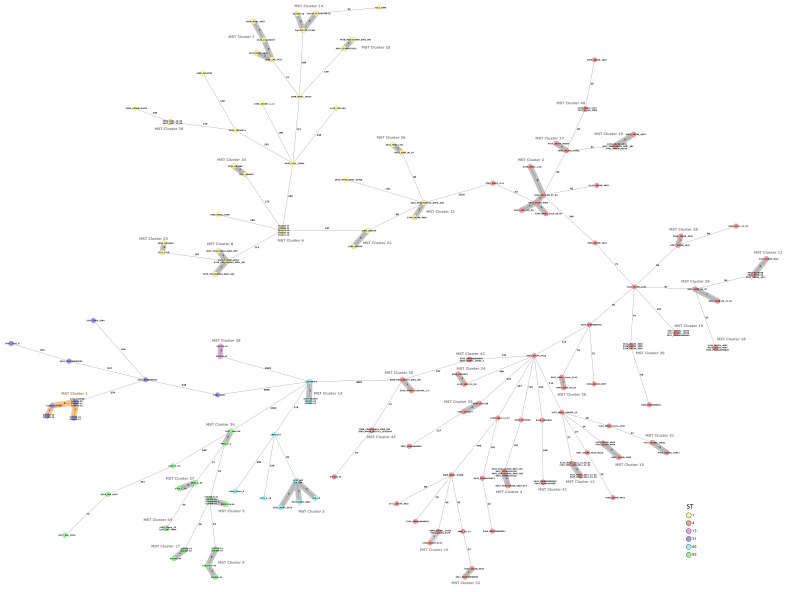
Minimum spanning tree (MST) of the 34 isolates analyzed in this study, incorporating an additional 143 *Cronobacter* isolates from the public PubMLST database. Isolates are represented as colored circles according to their sequence types (STs) as defined using the 7-loci MLST scheme. Black numbers on the connection lines indicate the number of allelic differences between isolates based on the cgMLST scheme, which comprises 2831 target genes for *Cronobacter sakazakii*/*C. malonaticus*. Isolates within a cluster threshold of 10 alleles are marked in grey as clusters.

After analyzing 174 additional strains from the public MLST database of *Cronobacter*, which included 32 strains of *C. sakazakii* ST1, 87 strains of ST4, 6 strains of ST31, 9 strains of ST83, and 9 strains of *C. malonaticus* ST60, it was observed that in cluster number 1 of ST31 strains, there was one closely related strain (3412_510556), which had been isolated from PIF in 2019 in Chile (Figure 2).

### 3.3. Antibiotic Susceptibility

All analyzed strains exhibited resistance to cephalothin (34/34). Resistance to ampicillin was also observed in 55.9% (19/34), to ceftazidime in 53% (18/34) of the strains, to amoxicillin–clavulanic acid in 14.7% (5/34), and to nalidixic acid in 5.8% (2/34). According to their antibiotic classes, these strains exhibited resistance to ampicillin (penicillins), amoxicillin–clavulanic acid (β-lactam combination agents), ceftazidime, cephalothin (cephalosporins), and nalidixic acid. Four strains were classified as multidrug-resistant (MDR), defined as resistant to three or more antibiotic classes. Notably, two ST1 strains were MDR, exhibiting resistance to four antibiotic classes (isolates 5101548 and 510175). Two strains (ST1 and ST31) were resistant to three antibiotic classes (isolates 510388 and 510414), and 14 isolates to two antibiotic classes. The MDR strains were primarily isolated from powdered milk, ingredients to prepare milk powder and milk powder mix (Supplementary Table S1).

Additionally, the three *C. malonaticus* strains were resistant to cephalothin, and one was resistant to ceftazidime (Supplementary Table S1).

### 3.4. Detection of Antibiotic Resistance and Virulence Genes

All analyzed strains possess β-lactamases with resistance to cephalothin on the bacterial chromosome. In *C. sakazakii* ST1, ST13, ST31, and ST83, *bla_CSA-1_* was detected, whereas *bla_CSA-2_* was found in ST4. In *C. malonaticus* ST60, *bla_CMA-1_* was identified. The *mcr-9*.*1* gene, conferring resistance to colistin, was detected in three strains of *C. sakazakii* ST1 (510292, 510175, and 5101548), all originating from powdered milk. The *dfrA51* gene (trimethoprim-resistant dihydrofolate reductase), conferring resistance to trimethoprim, was found in three strains of *C. sakazakii* ST83 (510364, 510363, and 510407) from powdered milk ingredients and dairy serum. The genes *qacG*, *qacJ*, *AcrAB-TolC* with *MarR* mutations, *adeF*, *marA*, *EF-tu*, and *msbA*, conferring resistance to disinfectants and various antibiotics, were found in 100% of the strains and are associated with resistance mechanisms such as antibiotic efflux, reduced permeability to antibiotics, and antibiotic target alteration.

Additionally, the heat resistance loci *hsp20* (small heat shock protein sHSP20), *clpK* (heat shock survival AAA family), *shsP* (small heat shock protein sHSP20-GI), *yfdx1* (heat resistance protein), *yfdx2* (heat resistance protein), *hdeD-GI* (heat resistance membrane protein), *trxLHR* (heat resistance system thioredoxin), *kefb-GI* (heat resistance system K+/H+ antiporter), and *psi-GI* (heat resistance protein) were identified on the chromosome of *C. sakazakii* ST4, while the genes *hsp20* and *clpK* were detected in all 12 strains of *C. sakazakii* ST83 (Table 2).

A total of 35 virulence genes were detected, with only 31 shared between *C. sakazakii* and *C. malonaticus* strains, as the *cpa* and *nanAKT* genes were present only in *C. sakazakii*. These genes were grouped into several categories: flagellar proteins (*flg*), outer membrane proteins (*ompA*), chemotaxis (*motB*), hemolysins (*hlyIII*), invasion (*lpxA*), plasminogen activator (*cpa*), colonization (*mviM*), small heat shock proteins (*ibpA*), transcriptional regulators (*sdiA*), macrophage survival, sialic acid usage (*nanA*,*K*,*T*), toxin–antitoxin systems (relB), and desiccation tolerance (wzzB) (Table 3).

### 3.5. Plasmids, Chromosome, and Mobile Genetic Elements

All strains exhibited plasmids of various origins. In *C. sakazakii* ST1 strains, the plasmid pESA3 (accession number CP000785) was detected in 100% of the analyzed strains. In *C. sakazakii* ST4, the plasmid pCS2 was identified, while the plasmid pSP291-1 was found in both isolates of ST13. Among the eight *C. sakazakii* ST31 strains, the plasmid pSP291-1 was only detected in two isolates, while the remaining six strains harbored the plasmid pCMA2 from *C. malonaticus*, with no differences according to the origin of the strains. Furthermore, 100% of the *C. sakazakii* ST83 isolates were found to carry the plasmid pSP291-2. Additionally, in three strains of ingredients for preparing PM (510364-24, 510363-24, and 510407-24), a smaller plasmid, pCS1, was detected. The three strains of *C. malonaticus* shared the plasmid pCMA1. Six unnamed plasmids were also identified which had previously been reported in *Escherichia coli* (4) and *Neisseria* (2). Eighty percent of the strains (27/34) exhibited mobile genetic elements (MGEs), with insertion sequences (ISs) being the most prevalent. To a lesser extent, composite transposons (cn) were identified in *C. sakazakii* ST83 strains, and a transposon (Tn) was found in one strain of *C. malonaticus* (530386-24) (Table 4).

## 4. Discussion

In 2022, a major manufacturer of powdered infant formula (PIF) initiated a voluntary recall due to intrinsic contamination following the emergence of four sporadic cases of invasive disease caused by *Cronobacter* spp. This situation led to a shortage of this vital food for several months, highlighting the importance of controlling this pathogenic microorganism in powdered dairy formulas [29].

In our study, 53% of the isolated strains were derived from powdered formulas or their ingredients, as well as from various manufacturing surfaces and environments. This aspect is relevant since powdered formulas are intrinsically non-sterile, and the product may become contaminated during production and subsequent rehydration. Furthermore, there is a potential risk of bacterial growth during reconstitution if basic factors such as the temperature of the rehydration water and holding time before consumption are not considered [30]. Ekundayo and Ijabadeniyi (2024) reported a global prevalence of *C. sakazakii* at 8.39%, with variation in powdered milk formulas depending on detection methods and differences in sample sizes used [31]. Controlling *Cronobacter* spp. in dairy product manufacturing begins with separating the external environment from the internal production process. Since this pathogen has been found on human skin and hands, it is essential to wash hands before entering the facility and to maintain good hygiene when changing clothes and shoes [32]. Additionally, contamination should be prevented through items such as work shoes, equipment, or wheeled carts, as they are efficient vectors for the pathogen’s spread in the environment [33], as demonstrated in our study where we found it in the soil and dust from control vacuum cleaners. Tong et al. (2024) [34], in their seven-year surveillance study in infant formula manufacturing plants, found that the source of *Cronobacter* spp. primarily originated from raw dry ingredients and the manufacturing environment, particularly from equipment such as vibrating sieves and vacuums. Additionally, in the combined process, contamination occurred in the packing room, where no subsequent thermal treatment was applied. Furthermore, it was observed that strengthening hygiene management regarding raw materials could help reduce the incidence of *Cronobacter* spp. in final products, such as powdered infant formula [34].

Core genome multilocus sequence typing (cgMLST) is crucial for distinguishing closely related species due to its high resolution and ability to identify subtle genetic differences. This method enhances epidemiological tracking, informs on transmission dynamics, and supports public health interventions effectively [35]. In our study, we utilized cgMLST for *C. sakazakii*/*C. malonaticus* to assess these closely related species and their temporal persistence. This scheme was employed in a multicenter study in Europe, which analyzed 59 isolates of *C. sakazakii* and three reference strains, revealing an average allelic difference of 2402 alleles. Moreover, eight isolates of *C. sakazakii* ST1, which included two epidemiologically related neonatal stool strains from Austria in 2009, showed only one allelic difference, highlighting the high discrimination power of the method [21]. In our analysis, we found that 33 out of 34 strains clustered into eight closely related clusters, regardless of the year and origin of the isolate, with the exception of *C. sakazakii* ST4. Previously, in 2021, a group of six ST1 strains of *C. sakazakii* was reported in Chile, exhibiting between one and three allelic differences, alongside one unrelated ST83 strain. In 2022, three closely related ST1 strains were identified [36]. In this regard, a strain of *C. sakazakii* ST31 isolated from powdered infant formula (PIF) in Chile in 2019 was closely related (no alleles of difference) to the ST31 strains from ingredients or environmental surfaces in this study, indicating it could be the primary source of contamination and persistence over time. The significance of this lies in the fact that *C. sakazakii* ST31 has been isolated from both PIF and clinical cases [37].

Infections caused by *C. sakazakii*, particularly those leading to septicemia and meningitis, require effective antibiotic treatments [38]. However, the resistance of strains in infant products is alarming, as consumers are often immunologically vulnerable. The inappropriate use of antibiotics in agriculture and livestock has increased multidrug resistance in these isolates [39]. In our study, all the strains examined were resistant to cephalothin and to a lesser extent to ceftazidime and ampicillin, with multidrug resistance (MDR) to four antibiotics (*C. sakazakii* ST1) and to three antibiotics classes (*C. sakazakii* ST1 and ST31). Caubilla-Barron et al. (2007) reported that strains isolated from two fatal neonatal infections expressed β-lactamases [40]. Hochel et al. (2012) found that all *Cronobacter* strains isolated from 399 retail food samples were resistant to erythromycin, and two of them were also resistant to tetracycline [41]. In Korea, Chon et al. (2012) reported that 77.8% of *Cronobacter* strains isolated from dehydrated foods were resistant to cephalothin [42]. Kilonzo-Nthenge et al. (2012) found that *C. sakazakii* isolates exhibited resistance to penicillin (76.1%), tetracycline (66.6%), ciprofloxacin (57.1%), and nalidixic acid (47.6%), while all were susceptible to gentamicin [43]. A study in China by Li et al. (2016) revealed high resistance to amoxicillin–clavulanate, rifampicin, tetracycline, streptomycin, and ampicillin in *C. sakazakii* strains isolated from milk-based infant foods [44]. Fei et al. (2017) also isolated *C. sakazakii* from PIF samples, finding high resistance to cephalothin [45]. Li et al. (2023) reported the presence of strains with 65% resistance to cephalothin [46]. In Iran, *C. sakazakii* strains resistant to ampicillin, amoxicillin, ciprofloxacin, and tetracycline were documented [39]. This resistance pattern was corroborated by Fei et al. (2022), who reported that 55.56% of the isolates were resistant to cephalothin and 96.30% to vancomycin [47]. Pakbin et al. (2022) [48] found that *C. sakazakii* isolates exhibited high resistance to several antibiotics, including amoxicillin–clavulanate (96%), amoxicillin (96%), ampicillin (96%), cefoxitin (92%), cefepime (92%), and others. Furthermore, 25 isolates were considered resistant to multiple drugs (MDR) [48]. Song et al. (2023), while analyzing 96 strains of *Cronobacter* spp., found that the strains of *C. sakazakii* and *C. malonaticus* were resistant to aminoglycosides, cephalosporins, and penicillins [49].

Muller et al. (2014) identified two unusual but very similar variants of the AmpC in *C. sakazakii* and *C. malonaticus* isolates, which conferred resistance exclusively to first-generation cephalosporins [50]. In this study the *AmpC* β-lactamases were designated *CSA-1* and *CSA-2* for *C. sakazakii* and *CMA-1* and *CMA-2* for *C. malonaticus*, those that we also found in this study. Specifically, *bla_CSA-1_* in *C. sakazakii* ST1, ST13, ST31, and ST83, while *bla_CSA-2_* was found in *C. sakazakii* ST4, and *bla_CMA-1_* was found in *C. malonaticus*. Other authors have also found resistance genes to these antibiotics in *Cronobacter* strains in China [51,52]. On the other hand, the colistin resistance gene was identified in three strains of *C. sakazakii* originating from PIF and PM. The *mcr-9*.*1* gene is considered a gene that can confer plasmid-mediated phenotypic resistance to colistin in various species of *Enterobacteriaceae*, which are public health concern pathogens. Colistin, or polymyxin, is an antibiotic with significant activity against Gram-negative bacteria, with the outer cell membrane being its primary site of action. This *mcr* gene circulates widely without detection unless induced by colistin [53,54,55]. Therefore, its presence constitutes a global concern, as colistin is regarded as a last-resort drug for treating infections caused by multidrug-resistant *Enterobacteriaceae* bacteria [56]; however, the presence of the gene in the genome of the bacteria does not imply that it is being expressed, which we corroborate with the phenotypic analysis, where no resistance to this antibiotic was detected. In addition, we found the *dfrA51* gene in three strains of *C. sakazakii* ST83 originating from PM ingredients, which confers resistance to trimethoprim. This is a new gene reported in 2025 in phage-plasmids, coding for proteins with at least 50% amino acid identity with all previously reported DfrA proteins [57]. Mobile *dfr* genes, which code for dihydrofolate reductases (DHFRs), are generally transported as cassettes in integrons or associated with insertion sequences (ISs). They have been identified in enterobacteria such as *Salmonella* and *E*. *coli*, which are pathogens associated with infections in humans [58,59].

In our study, a similar virulence gene profile was found in both *C. sakazakii* and *C. malonaticus*. Differences were only noted in *C. malonaticus*, as the *cpa* and *nanAKT* genes were not present. Joseph et al. (2013) [60] report that the *nanAKT* gene encodes for the utilization of sialic acid, a substance that occurs naturally in breast milk and is artificially added to infant formulas to promote brain development in children. Therefore, the use of sialic acid by *C. sakazakii* as a carbon source for its growth and proliferation in the host makes it a potential health risk for those consuming these foods [60]. Additionally, the Cpa protein is linked to serum resistance and systemic spread of *C. sakazakii*. The *cpa* locus could be considered specific to *C. sakazakii* and *C. universalis* [61]. However, clinical strains of *C. sakazakii* of type ST8 have been identified, which are highly virulent and harbor the pESA3 plasmid yet lack the *cpa* gene. This suggests the likely existence of other virulence genes responsible for the disease [62] or that the *cpa* gene may be encoded in the chromosome, as observed in our study in two *C. sakazakii* ST31 strains whose plasmid is derived from *C. malonaticus*. The *C. malonaticus* plasmid pCMA2 found in the *C. sakazakii* ST31 strains contained the *arsABCD* cassette, which encodes the arsenate reductase enzyme and efflux pumps, among others. These features enable microorganisms to survive in environments contaminated with arsenic by reducing this toxin intracellularly. Additionally, cross-resistance to antibiotics and heavy metals was observed [63,64]. Furthermore, copper resistance genes were found, which allow pathogens to survive in hostile environments, considering that this metal is widely used in food processing plants as a bactericide [65].

On the other hand, genes associated with heat shock proteins, such as Hsp20, have been reported in thermotolerant strains of *C. sakazakii* [66,67]. Therefore, when these *C. sakazakii* strains isolated from PIF and PM production environments exhibit thermal resistance loci or genes, as seen in our study (*C. sakazakii* ST4 and ST83), they acquire greater resilience that enables them to persist in production environments and in food [68]. This is confirmed by the study of Myintzaw et al. (2026) [69], which characterized the capacity of *C. sakazakii* ST1, ST4, and ST8 to tolerate high temperatures (90 °C) in a commercial PIF matrix. They demonstrated that thermotolerance is associated with the sequence type of *C. sakazakii*, with ST4 being the most tolerant due to the presence of heat shock proteins, thermal resistance genes and fimbrial chaperones, which have also been reported in this study [69]. This suggests that recommendations such as using rehydration water at 70 °C may not be sufficient to ensure the safety of the final product [70].

In our study, strains of *Cronobacter* spp. (*C. sakazakii* and *C. malonaticus*) were identified from both powdered milk and surfaces and environments within a formula manufacturing plant. Additionally, antibiotic resistance and virulence genes were identified, which not only increase the risk of infections but also affect the severity and outcomes of the disease. All these aspects are essential for reviewing and improving hygiene practices in the locations where dairy formulas are produced.

## 5. Conclusions

The isolates of *C. sakazakii* and *C. malonaticus* characterized in this study demonstrated resistance to multiple antibiotics, as well as the presence of various antibiotic resistance genes, thermal resistance genes, and virulence factors. Therefore, powdered dairy products contaminated with *C. sakazakii* pose a significant risk to consumer health. Thus, strict adherence to good manufacturing practices and hygiene in plants is necessary, along with a review of the recommendations regarding the rehydration water temperature for powdered milk formulas. Additionally, multicenter studies are required due to the consumption of these infant formulas in various countries.

## Figures and Tables

**Table 1:** Identification of *Cronobacter* spp. strains isolated from different sources by rMLST and whole genome sequencing.

Sample ID	Source *	Collection Date	rMLST	ST	CC	Serotype (*galF-gnd* Alleles)
510413-19	Milk powder ingredient	2017	*C. sakazakii*	1	1	*Csak O1*
510360-24	Milk powder ingredient	2017	*C. sakazakii*	1	1	*Csak O1*
5101548-22	Powdered milk	2022	*C. sakazakii*	1	1	*Csak O1*
510388-24	Milk powder ingredient	2015	*C. sakazakii*	1	1	*Csak O1*
510427-19	Powdered milk	2017	*C. sakazakii*	1	1	*Csak O1*
510292-18	PIF	2017	*C. sakazakii*	1	1	*Csak O1*
510426-19	Powdered milk	2017	*C. sakazakii*	1	1	*Csak O1*
510175-22	Powdered milk	2022	*C. sakazakii*	1	1	ND
510415-19	PIF	2011	*C. sakazakii*	4	4	*Csak O4*
510411-19	Milk powder ingredient	2018	*C. sakazakii*	13	13	*Csak O2*
510378-24	PIF	2015	*C. sakazakii*	13	13	*Csak O2*
510387-24	Milk powder ingredient	2016	*C. sakazakii*	31	31	*Csak O2*
510414-19	Milk powder mix	2016	*C. sakazakii*	31	31	*Csak O2*
510409-19	Vacuum cleaner dust	2017	*C. sakazakii*	31	31	*Csak O2*
510365-24	Dairy company floor	2016	*C. sakazakii*	31	31	*Csak O2*
510406-19	Dairy company floor	2016	*C. sakazakii*	31	31	*Csak O2*
510374-24	Vacuum cleaner dust	2016	*C. sakazakii*	31	31	*Csak O2*
510431-19	Vacuum cleaner dust	2019	*C. sakazakii*	31	31	*Csak O2*
510369-24	Vacuum cleaner dust	2016	*C. sakazakii*	31	31	*Csak O2*
510371-B-24	Vacuum cleaner dust	2016	*C. sakazakii*	83	83	*Csak O7*
510405-19	Vacuum cleaner dust	2016	*C. sakazakii*	83	83	*Csak O7*
510370-24	Vacuum cleaner dust	2016	*C. sakazakii*	83	83	*Csak O7*
510402-19	Vacuum cleaner dust	2016	*C. sakazakii*	83	83	*Csak O7*
510408-19	Vacuum cleaner dust	2017	*C. sakazakii*	83	83	*Csak O7*
510368-B-24	Vacuum cleaner dust	2016	*C. sakazakii*	83	83	*Csak O7*
510364-24	Milk powder ingredient	2016	*C. sakazakii*	83	83	*Csak O7*
510407-19	Milk serum	2016	*C. sakazakii*	83	83	*Csak O7*
510403-19	Vacuum cleaner dust	2016	*C. sakazakii*	83	83	*Csak O7*
510363-24	Milk powder ingredient	2016	*C. sakazakii*	83	83	*Csak O7*
510377-A-24	PIF	2018	*C. sakazakii*	83	83	*Csak O7*
510366-24	PIF	2018	*C. sakazakii*	83	83	*Csak O7*
510375-24	Vacuum cleaner dust	2015	*C. malonaticus*	60	60	*Cmal O1*
510380-24	Vacuum cleaner dust	2016	*C. malonaticus*	60	60	*Cmal O1*
510386-24	Vacuum cleaner dust	2016	*C. malonaticus*	60	60	*Cmal O1*

rMLST: Ribosomal multilocus sequence typing; ST: Sequence type; CC: clonal complex; ND: not determined. * Vacuum cleaners had been used to clean equipment in dairy environments.

**Table 2:** Antibiotic-resistant genes identified by AMRFinderPlus and Comprehensive Antibiotic Resistance Database (CARD) of *C. sakazakii* and *C. malonaticus* strains.

ST	Species	Antibiotic Resistance	Resistance Genes
1	*C. sakazakii*	*AM*, *CAZ*, *KF*, *W*	*bla_CSA-1_*, *mcr-9*.*1*, *qacG*, *AcrAB-TolC* with *MarR mutations*, *adeF*, *marA*, *EF-tu*, *msbA*
4	*C. sakazakii*	*AM*, *KF*	*bla_CSA-2_*, *qacG*, *AcrAB-TolC* with *MarR mutations*, *adeF*, *hsp20*, *clpK*, *shsP*, *yfdx1*, *yfdx2*, *hdeD-GI*, *trxLHR*, *kefb-GI*, *psi-GI*
13	*C. sakazakii*	*KF*	*bla_CSA-1_*, *qacG*, *adeF*, *AcrAB-TolC* with *MarR mutation*, *marA*, *qacG*
31	*C. sakazakii*	*AM*, *AMC*, *CAZ*, *KF*	*bla_CSA-1_*, *qacG*, *adeF*, *AcrAB-TolC* with *MarR mutation*, *marA*
83	*C. sakazakii*	*AMP*, *CAZ*, *KF*	*bla_CSA-1_*, *dfrA51*, *qacJ*, *adeF*, *AcrAB-TolC * with *MarR mutation*, *marA*, *hsp20*, *clpK*
60	*C. malonaticus*	*CAZ*, *KF*	*bla_CMA-1_*, *qacG*, *adeF*, *AcrAB-TolC* with *MarR mutation*, *marA*

*AM*: ampicillin; *AMC*: amoxicillin–clavulanic acid; *CAZ*: Ceftazidime; *KF*: cephalothin; *W*: nalidixic acid.

**Table 3:** Putative virulence gene distribution in strains of *Cronobacter sakazakii* and *Cronobacter malonaticus* according to sequence type (ST).

Virulence Gene	Function	*C*. *sakazakii* ST1	*C*. *sakazakii* ST4	*C*. *sakazakii* ST13	*C*. *sakazakii* ST31	*C*. *sakazakii* ST83	*C*. *malonaticus* ST60
*flgB*	motility	+	+	+	+	+	+
*flgK*	flagellar hook-associated protein 1	+	+	+	+	+	-
*flgL*	flagellar hook-associated protein 3	+	+	+	+	+	+
*flgM*	negative regulator of flagellin synthesis	+	+	+	+	+	+
*flgN*	flagella synthesis FlgN protein	+	+	+	+	+	+
*flhD*	flagellar hook-associated protein 2	+	+	+	+	+	+
*fliA*	flagellar operon FliA	+	+	+	+	+	+
*fliC*	flagellin	+	+	+	+	+	+
*fliD*	flagellar hook-associated protein 2	+	+	+	+	+	+
*fliR*	flagellar biosynthetic FliR protein	+	+	+	+	+	+
*fliT*	flagella FliT protein	+	+	+	+	+	+
*fliZ*	FliZ protein	+	+	+	+	+	+
*lolA*	outer membrane lipoprotein carrier protein	+	+	+	+	+	+
*motB*	chemotaxis MotA protein	+	+	+	+	+	+
*sdiA*	LuxR family transcriptional regulator	+	+	+	+	+	+
*slyB*	outer membrane lipoprotein SlyB	+	+	+	+	+	+
*tolC*	outer membrane channel protein	+	+	+	+	+	+
*msbA*	survival in macrophage	+	+	+	+	+	+
*mviN*	protective immunity and colonization	+	+	+	+	+	+
*cpa*	plasminogen activator	+	+	+	+	+	-
*Hha*	hemolysin expression modulating protein	+	+	+	+	+	+
*hly III*	hemolysin III	+	+	+	+	+	+
*ompA*	adhesion cell; biofilm formation	+	+	+	+	+	+
*ompX*	adhesion cell	+	+	+	+	+	+
*Blc*	outer membrane lipoprotein	+	+	+	+	+	+
*cheR*	chemotaxis protein methyltransferase	+	+	-	+	+	+
*cheY*	response regulator of chemotaxis family	+	+	+	+	+	+
*lpxA*	epithelial cell invasion and lipid A production	+	+	+	+	+	+
*nanA*,*K*,*T*	utilization of exogenous sialic acid	+	+	+	+	+	-
*Fic*	cell filamentation protein	+	+	+	+	+	+
*relB*	antitoxin to RelE	+	+	+	+	+	+
*ibpA*	small heat shock protein *IbpA*	+	+	+	+	+	+
*ibpB*	small heat shock protein *IbpB*	+	+	+	+	+	+
*hspQ*	encoding proteases heat	+	+	+	+	+	+
*wzzB*	desiccation tolerance	+	-	+	+	+	+

+ = presence; - = absence. *flgB*: flagellar basal body rod protein; *flgK*: flagellar hook-associated protein 1; *flgL*: flagellar hook-associated protein 3; *flgM*: anti-sigma factor for FliA; *flgN*: Flagella synthesis protein FlgN; *flhD*: Flagellar transcriptional activator FlhD; *fliA*: flagellin-specific sigma factor; *fliC*: flagellin; *fliD*: flagellar hook-associated protein 2; *fliR*: flagellar biosynthetic protein FliR; *fliT*: flagellar biosynthesis protein T; *fliZ*: DNA-binding transcriptional regulator FliZ; *lolA*: periplasmic chaperon for lipoproteins; *motB*: motility protein B; *sdiA*: suppressor of division inhibition A; *slyB*: outer membrane lipoprotein SlyB; *tolC*: outer membrane protein TolC; *msbA*: ATP-dependent lipid A-core flippase; *mviN*: mouse virulence gene N; *cpa*: *Cronobacter* plasminogen activator; *Hha*: Hemolysin expression-modulating protein Hha; *hly III*: Hemolysin III; *ompA*: outer membrane protein A gene; *ompX*: outer membrane protein X; *blc*: outer membrane lipoprotein Blc; cheR: chemotaxis protein methyltransferase CheR; cheY: chemotaxis protein CheY; lpxA: Acyl-[acyl-carrier-protein]--UDP-N-acetylglucosamine O-acyltransferase: *nanA*,*K*,*T*: *nanA* (N-acetylneuraminate lyase), *nanK* (N-acetylmannosamine kinase), and *nanT* (sialic acid transporter); *fic*: filamentation induced by cAMP-family toxins; *relB*: antitoxin coding gene; *ibpA*: inclusion body-binding protein A (sHsp); *ibpB*: inclusion body-binding protein B (sHsp); *hspQ:* heat shock protein hspQ; *wzzB*: polisoprenol-linked O-antigen transporter.

**Table 4:** Plasmids and mobile genetic elements of *Cronobacter sakazakii* and *Cronobacter malonaticus* strains.

ID Strain	Species	ST	Plasmid	Accession Number	Rep Types	Size (Kb)	Mobile Genetic Elements
510413-19	*C. sakazakii*	1	p131.0_510413-9	CP000785	IncFIB, rep_cluster_574	130,955	NDT
			p23.1_510413-19	FN543095	IncFIB, IncFII	23,137	
510360-24	*C. sakazakii*	1	p130.2_510360-24	CP000785	IncFIB, rep_cluster_574	130,206	NDT
			p23.1_610360-24	FN543095	IncFIB, IncFII	23,087	
5101548-22	*C. sakazakii*	1	p131.7_5101548_22	CP000785	InFIB, rep_cluster_574	131,696	ISKpn74, IS903, ISPpu12, ISEsa2, ISEsa1, IS5075, ISSen9
			p18.1_5101548-22	CP035364	ND	18,088	
			p3.8_5101548-22	KM373703	rep_cluster_2335	3845	
510388-24	*C. sakazakii*	1	p134.2_510388-24	CP027110	InFIB, rep_cluster_574	134,192	NDT
			p23.1_510388-24	FN543095	IncFIB, IncFII	23,087	
510427-19	*C. sakazakii*	1	p130.7_510427-19	CP000785	InFIB, rep_cluster_574	130,682	NDT
			p23.1_510427-19	FN543095	IncFIB, IncFII	23,137	
510292-18	*C. sakazakii*	1	p131.4_510292-18	CN000785	InFIB, rep_cluster_574	131,422	cn_1030_ISSen9, ISSen9, ISPpu12, IS903, IS26, ISKpn74, IS5075, ISEsasa1, ISEsa2
			p14.6_510292-18	CP035346	ND	14,632	
			p11.9_510292-18	CP035364	ND	11,948	
			p4.0_510292-18	KM373703	rep_cluster_2335	3959	
510426-19	*C. sakazakii*	1	p130.7_510426-19	CP000785	InFIB, rep_cluster_574	130,696	NDT
			p23.1_510426-19	FSN43095	IncFIB, IncFII	23,137	
510175-22	*C. sakazakii*	1	p130.2_510175-22	CP000785	InFIB, rep_cluster_574	130,197	ISEsa2, ISEsa1, IS102, IS903, IS5075, ISSen9, ISPpu12, IS26, cn_861_ISSen9
510415-19	*C. sakazakii*	4	p49.8_510415-19	CP012255	ND	49,763	ISEhe3, ISEsa2
510411-19	*C. sakazakii*	13	p114.7_510411-19	CP004092	IncFIB, rep_cluster574	114,746	ISSen9
			p102.7_510411-19	CP012254	rep_cluster_763	102,689	
			p43.9_510411-19	CP013942	IncFIB	43,934	
510378-24	*C. sakazakii*	13	p111.9_510378-24	CP004092	IncFIB, rep_cluster574	111,870	ISSen9
			p84.2_510378-24	CP012254	rep_cluster_763	84,167	
			p42.5_510378-24	CP013942	IncFIB	42,472	
510387-24	*C. sakazakii*	31	p116.0_510387-24	CP004092	IncFIB, rep_cluster574	116,026	ISEsa1
			p55.8_510387-24	CP013942 (pCMA2)	ND	55,836	
510414-19	*C. sakazakii*	31	p55.9_510414-19	CP013942 (pCMA2)	ND	55,886	ISEsa1
			p1.2_510414-19	LT592160 (WHO_U)	ND	1219	
510409-19	*C. sakazakii*	31	p55.9_510409-19	CP013942 (pCMA2)	ND	55,886	ISEsa1
			p1.2_510409-19	CP034021 (FQ48)	ND	1201	
510365-24	*C. sakazakii*	31	p55.8_510365-24	CP013942 (pCMA2)	ND	55,836	ISEsa1
510406-19	*C. sakazakii*	31	p55.9_510406-19	CP013942 (pCMA2)	ND	55,886	ISEsa1
			p1.1_510406-19	CP034021 (FQ48)	ND	1091	
510374-24	*C. sakazakii*	31	p114.6_510374-24	CP004092	IncFIB, rep_cluster574	114,631	IS481, ISEsa1
			p34.7_510374-24	CP011050	ND	34,663	
			p16.4_510374-24	CP035364	ND	16,380	
510431-19	*C. sakazakii*	31	p55.9_510431-19	CP013942 (pCMA2)	ND	55,886	ISEsa1
510369-24	*C. sakazakii*	31	p55.8_510369-24	CP013942 (pCMA2)	ND	55,836	ISEsa1
510371-24	*C. sakazakii*	83	p118.3_510371-24	CP004092	IncFIB, rep_cluster574	118,275	
510405-19	*C. sakazakii*	83	p118.3_510405-19	CP004092	IncFIB, rep_cluster574	118,325	cn_10824_ISSen9, ISSen9
510370-24	*C. sakazakii*	83	p118.2_510370-24	CP004092	IncFIB, rep_cluster574	118,177	ISSen9
510402-19	*C. sakazakii*	83	p118.3_510402-19	CP004092	IncFIB, rep_cluster574	118,325	cn_10824_ISSen9, ISSen9
510408-19	*C. sakazakii*	83	p118.3_510408-19	CP004092	IncFIB, rep_cluster574	118,325	cn_10824_ISSen9, ISSen9
510368-24	*C. sakazakii*	83	p118.2_510368_24	CP004092	IncFIB, rep_cluster574	118,223	cn_10824_ISSen9, ISSen9, IS481
510364-24	*C. sakazakii*	83	p118.2_510364-24	CP004092	IncFIB, rep_cluster574	118,175	cn_10824_ISSen9, ISEsa2, ISEsa1, IS481
			p92.7_510364-24	CP012254 (*dfrA51*)	rep_cluster_763	92,736	
510407-19	*C. sakazakii*	83	p118.3_510407-19	CP004092	IncFIB, rep_cluster574	118,325	ISEsa1, ISEsa2, ISSen9
			p92.8_510407-19	CP012254 (*dfrA51*)	rep_cluster_763	92,821	
510403-19	*C. sakazakii*	83	p118.3_510403-19	CP004092	IncFIB, rep_cluster574	118,325	ISSen9
510363-24	*C. sakazakii*	83	p118.2_510363-24	CP004092	IncFIB, rep_cluster574	118,196	cn_10824_ISSen9, ISEsa1, ISEsa2
			p99.6_510363-24	CP012254 (*dfrA51*)	rep_cluster_763	99,571	
510377-24	*C. sakazakii*	83	p118.2_510377-24	CP004092	IncFIB, rep_cluster574	118,158	cn_10824_ISSen9, ISSen9
510366-24	*C. sakazakii*	83	p118.3_510366-24	CP004092	IncFIB, rep_cluster574	118,275	ISSen9
510375-24	*C. malonaticus*	60	p128.5_510375-24	CP013941 (pCMA1)	IncFIB, rep_cluster574	124,494	NDT
			p42.2_510375-24	CP035346	ND	42,223	
510380-24	*C. malonaticus*	60	p113.4_510380-24	CP013941	IncFIB, rep_cluster574	113,363	NDT
			p17.0_510380-24	CP035364	ND	16,965	
			p4.7_510380-24	CP035346	ND	4691	
510386-24	*C. malonaticus*	60	p135.2_510386-24	CP013941	IncFIB, rep_cluster574	135,244	Tn6024

ND: not determined; NDT: not detected.

## Data Availability

The original contributions presented in this study are included in the article/Supplementary Materials. Further inquiries can be directed to the corresponding authors.

## Supplementary Materials

The following supporting information can be downloaded at: https://www.mdpi.com/article/10.3390/microorganisms14030593/s1, Supplementary Table S1: Resistance Profile.
